# Does health early at arrival predict later integration among refugees? A cohort study of Syrians in Norway

**DOI:** 10.1186/s12939-026-02837-8

**Published:** 2026-04-07

**Authors:** Yeneabeba Tilahun Sima, Elisabeth Marie Strømme, Astrid Lunde, Esperanza Diaz

**Affiliations:** Present Address: Department of Global Public Health and Primary Care, University of Bergen, P.O. BOX 7804, Bergen, 5020 Norway

**Keywords:** Health, Integration, Cohort study, Refugees, Norway

## Abstract

**Background:**

Integration of refugees in host countries is high on the political agenda, but the prospective influence of health at arrival on later integration outcomes remains unclear. This study describes the integration of Syrian refugees in Norway four years post-resettlement, exploring whether their health in early resettlement period serves as a predictor of subsequent integration.

**Methods:**

This prospective cohort study used data from the CHART/Integration for Health project, which examines the intertwined development of health and integration among Syrian quota refugees living in Norway four years after recruitment in Lebanon. Health status was assessed one-year post-arrival through self-reported chronic pain, non-communicable diseases (NCDs), anxiety/depression, and post-traumatic stress symptoms. Integration was measured four years after resettlement using the Immigration Policy Lab (IPL) Integration Index, with scores normalized from 0 to 1, where higher values indicate greater integration. Hierarchical linear regression was used to examine whether early post-resettlement health predicted overall and dimension-specific integration, adjusting for age, gender, and educational attainment.

**Results:**

Four years after resettlement, Syrian refugees reported difficulties across several integration domains particularly social- and linguistic integration. Those reporting chronic pain were more likely to face challenges in economic integration (β = − 0.24, *p* < 0.05), while refugees with NCDs encountered greater difficulties in linguistic integration (β = − 0.17, *p* < 0.01). Additionally, symptoms of anxiety/depression one year after arrival were associated with later increased overall integration challenges, particularly navigating services (β = − 0.42, *p* < 0.01). However, poorer health did not uniformly predict disadvantage and was occasionally associated with fewer challenges in specific areas of integration.

**Conclusion:**

Poor health during the early post-resettlement period was mostly linked to greater challenges across multiple integration domains four years later. These findings underscore the importance of addressing refugees´ health needs shortly after arrival, within the broader context of integration. Ensuring timely and accessible healthcare at early resettlement staged, alongside comprehensive integration efforts, could promote better and more equitable integration outcomes for refugees.

**Supplementary Information:**

The online version contains supplementary material available at 10.1186/s12939-026-02837-8.

## Background

The global refugee crisis continues to escalate, with Europe hosting over one-third of the world’s refugees as of 2023 [1]. In Norway, the refugee population now exceeds 333,500 individuals, accounting for about 6% of the total population and 35% of all immigrants [2]. The prolonged Syrian conflict has significantly reshaped Norway’s refugee demographics, making Syrians the largest non-European refugee group both nationally and across Europe [3]. Upon arrival, Syrian refugees show higher rates of anxiety (30%), depression (45%), post-traumatic stress disorder (PTSD) (30%), and chronic pain (43%) compared to the general population [4]. While resettlement provides safety, adapting to new cultural, linguistic, and social environments often worsens these health challenges [5], resulting in increased health needs that affect quality of life [6] and raise concerns about health equity [7]. Addressing these intertwined health and settlement challenges is crucial for supporting integration into Norwegian society.

With the growing number of refugees and the substantial personal and societal costs tied to resettlement, integration has become a key policy priority in Norway [8]. Contrary to assimilation, which entails the abandonment of one’s cultural identity, integration is often viewed as a reciprocal process that encourages refugees’ social, economic, and cultural participation while the host society acknowledges and respects their identities [9]. This mutual engagement establishes shared responsibilities: refugees contribute to their new communities, and host societies create inclusive and supportive environments. However, the lack of a universally accepted definition, combined with the diverse political, social, and cultural contexts across resettlement settings, complicates the development of standardized frameworks [10]. As a result, integration is increasingly understood as a multifaceted, context-specific process that requires adaptable and nuanced approaches reflecting the experiences of both refugees and host communities [11].

The integration of Syrian refugees resettled in many European countries has typically been evaluated using conventional approaches that focus on macro-level indicators such as employment, income and education [12–14]. While these metrics remain vital, they capture only part of the integration process. There is growing scholarly consensus on the need to incorporate subjective and relational dimensions, like sense of belonging, social networks, and perceived acceptance, to better represent the full complexity of integration experiences [11]. Recognizing this multidimensionality, the present study adopts the framework proposed by Harder and colleagues, which conceptualizes integration across six interconnected domains: psychological, economic, political, social, linguistic, and navigational [15]. Within this model, successful integration is defined as achieving high levels of functioning across all dimensions, reflecting balanced progress and active participation in multiple aspects of life.

Health is not only a crucial marker of integration success but also a fundamental resource that enables refugees to participate meaningfully in their new communities [10]. Previous research has demonstrated strong links between health difficulties and challenges in integration [5, 13, 16, 17]. For instance, studies from the Netherlands indicate that refugees experiencing health problems are more likely to be unemployed and occupy lower occupational positions [13]. Similarly, evidence from Switzerland shows that refugees with severe PTSD exhibit poorer integration outcomes [17]. However, much of this evidence comes from cross-sectional studies conducted years after resettlement, limiting insights into how health at the early stages can impact other facets of integration. This is a critical gap, as early health status can impact refugees’ capacity to engage with their new communities [18]. Consequently, the early resettlement period offers a crucial window for cross-sectoral policy interventions to maintain and improve health [19, 20]. Moreover, while mental health has received substantial research attention [5, 16, 17], the role of physical health in shaping integration remains underexplored.

Despite Norway’s universal healthcare system and generous welfare provisions, refugees in Norway continue to face challenges accessing healthcare after resettlement [21]. Although refuges are legally entitled to publicly funded healthcare services with minimal fees [22] and benefit from the mandatory Introduction Programme, which offers language training, education, employment support, and community engagement opportunities [23], access to healthcare remains uneven [21, 24]. While Syrian refugees initially experienced some health improvements [6, 20], studies indicate that their health deteriorates over time [25]. Refugees demonstrate patterns of both overuse and underuse of healthcare services [25–29], reflecting persistent inequalities driven by a combination of individual and systemic factors [21]. These disparities can perpetuate ongoing health challenges and deepen social and economic inequalities [30].

Much of the existing evidence on refugee health and integration is based on cross-sectional studies conducted several years after resettlement, offering limited insight into how health at an early stage of post-resettlement relates to later integration. To address this gap, we use longitudinal data from Syrian refugees, the second-largest refugee population in Norway [3], to examine whether health measured one year after arrival is associated with integration outcomes four years later. This study identifies subgroups within the Syrian refugee population in Norway with physical and mental health challenges who may benefit from earlier and more targeted support, thereby informing interventions aimed at promoting more equitable and sustainable integration trajectories.

### Conceptual framework

**Fig. 1:**
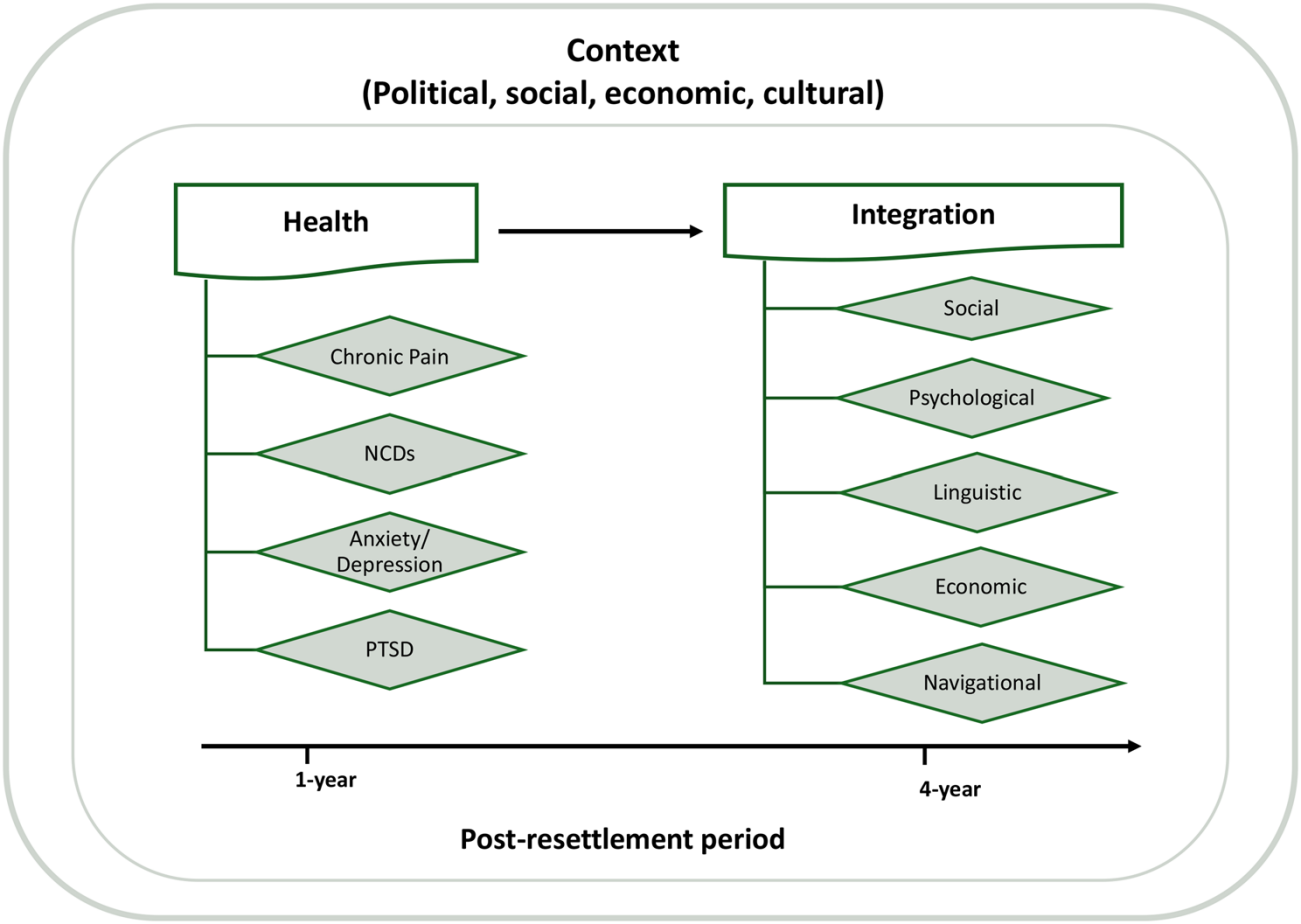
Conceptual framework of the role of health in the early post-resettlement period and later refugee integration. NCDs - Non-communicable disease. PTSD - Post-traumatic stress disorders

This study uses the Ager and Strang (2008) Framework of Integration to understand the relationship between health and integration in the Norwegian context [10]. The framework identifies ten core domains that shape refugee integration and is well suited to this study because it treats health not only as a *marker* of integration, but also as a *means* that enables participation across other domains. Poor health can therefore limit refugees’ ability to engage with and adapt to their host society (Fig. 1).

Integration is defined as the degree to which migrants develop the skills, understanding, and opportunities needed to build a meaningful life in the host society [15]. In this study, we measured integration using the Immigration Policy Lab Index (IPL-12/24), which identifies six key dimensions of support essential for refugees to establish stable and fulfilling lives. Unlike measures that focus on individual preferences [31, 32], the IPL Index focuses on refugees’ current social ties and engagement within the host community, offering a comprehensive metric aligned with this study goals.

## Methods

### Study design, settings and data collection

This prospective cohort study is part of the Integration for Health (I4H) project, which is a continuation from the Changing Health and health care needs Along the Syrian Refugees’ Trajectories to Norway (CHART) project. These projects aim to assess the health and health needs Syrian refugees along their resettlement journey [20, 33].

All Syrian refugees aged 16 years and older attending the mandatory pre-departure orientation in Lebanon were invited to participate in the survey. A total of 506 refugees (Wave-1) completed a self-administered Arabic questionnaire between August 2017 and April 2018, with trained staff available to assist as needed. Participants were then invited to a follow-up telephone survey one year after their resettlement in Norway (December 2018 to December 2019). Contact information was obtained from the Norwegian Directorate of Integration and Diversity and public refugee offices. Of the 465 refugees who were successfully reached, 353 (76%) completed the interview (Wave-2). The same group was invited to a third round of phone interviews four years after resettlement (August 2022 to December 2023). Of the 464 individuals invited, 153 (33%) participated (Wave-3). For both follow-up surveys, questionnaires were administered over the phone and conducted in Arabic by Arabic-speaking staff. Loss to follow-up was mainly due to unsuccessful contact after three call attempts or refusal to participate.

**Fig. 2:**
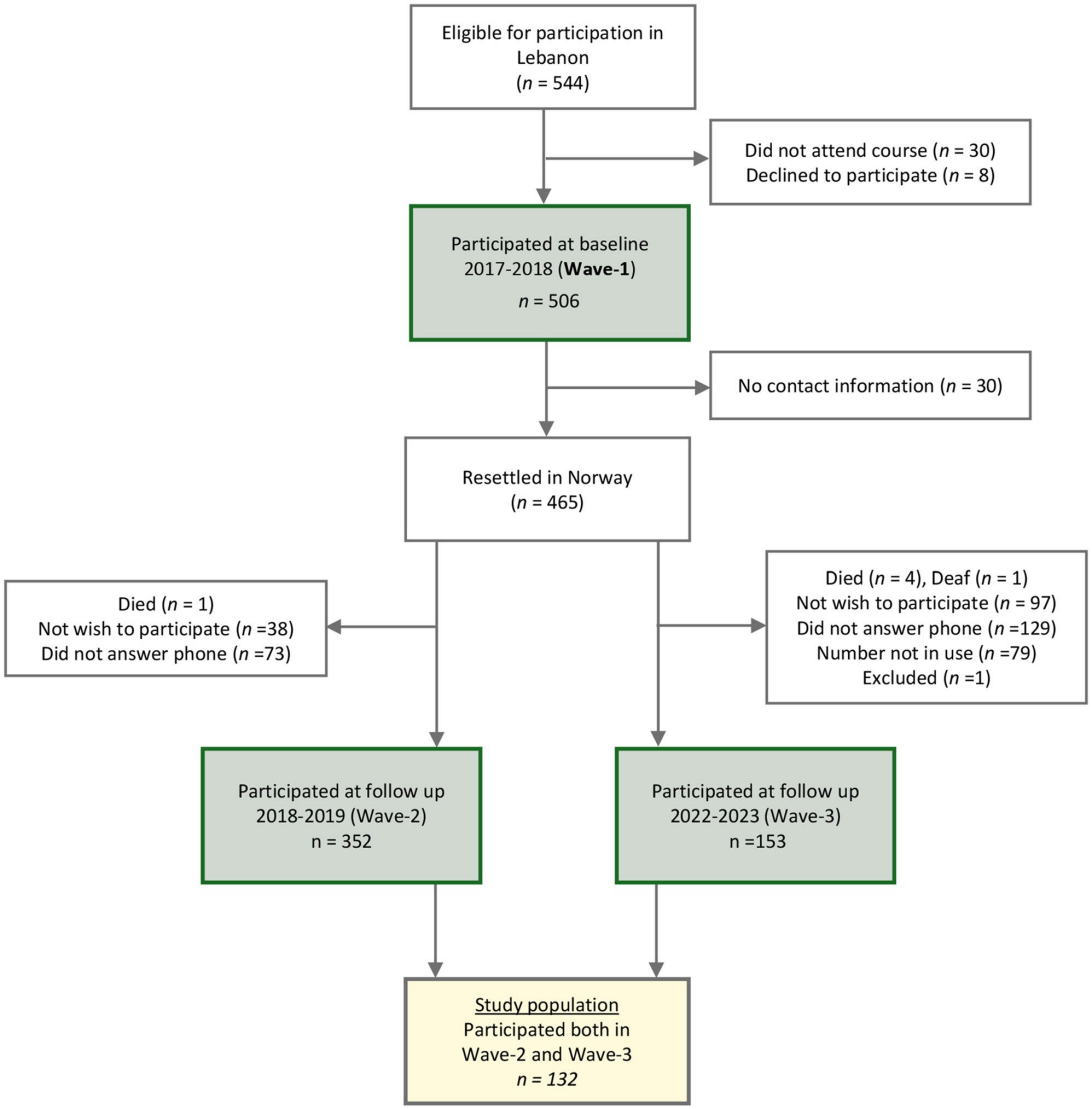
Flowchart of the study population

The study included participants who completed both Wave-2 and Wave-3 surveys (Fig. 2).

### Ethical approval

The study was approved by the Regional Committee for Medical and Health Research Ethics in Southeast Norway (ref. no. 2017/377). Participants provided written consent at baseline and verbal consent at follow-up surveys.

### Variables and measures

#### Dependent variables

Integration was evaluated using the Immigration Policy Lab (IPL-12) questionnaire, a validated instrument designed to capture multidimensional aspects of integration across varied settings, including Norway [5, 15, 16]. In the present study, only five domains were included, with political integration excluded to minimise potential participant discomfort.

Social integration was measured using two items assessing the frequency of contact with Norwegians. Participants were asked how often they shared a meal or coffee with Norwegians (1 = “never” to 5 = “very often”) and how many phone contacts they had with Norwegians in the past four weeks (0 to “15 or more”). The internal consistency of this index was acceptable (α = 0.60). Psychological integration was assessed with four items: “How connected do you feel with Norway?” (1 = not at all, 5 = extremely close); “Thinking about your future, where do you want to live?” (1 = definitely want to move to another country, 5 = definitely in Norway); “How often do you feel isolated from Norwegian society?”; and “How often do you feel like an outsider in Norway?” (1 = always, 5 = never). Internal consistency was acceptable (α = 0.61). Linguistic integration was based on two items rating Norwegian reading and speaking skills, with scores from 1 (“not well”) to 5 (“very well”), showing good internal consistency (α = 0.79).

Economic integration was measured using employment status only, as combining employment and income items resulted in low internal consistency (α = 0.20). Although income is an important indicator, employment was chosen because it more directly reflects an individual’s ability to navigate the system and secure work, which are key components of post-resettlement integration [10]. Employment status was categorized on a scale from 1 to 5: 1 = unemployed and not seeking work, 2 = unemployed but actively seeking work, 3 = retired or disabled, 4 = student or participating in the introduction program, and 5 = employed. Navigational integration was initially assessed using two IPL-12 items on the difficulty of (a) seeing a doctor and (b) job searching in Norway. Due to low internal consistency (α = 0.36), only the item on seeing a doctor was retained, as it was more relevant to this study. Responses ranged from 1 (“very difficult”) to 5 (“very easy”).

Scores for each integration dimension were computed by summing the relevant item responses. An overall integration score was then derived by summing all 10 items across the five domains. Individual domain score and the overall integration score were subsequently normalized to a 0–1 scale, with higher values indicating greater levels of integration.

#### Independent variables

The health status of Syrian refugees reported one year after their arrival in Norway (Wave-2), was the exposure. Poor health is defined as having at least one of the following self-reported conditions: chronic pain, a non-communicable disease (NCD), symptoms of anxiety/depression, or PTSD. Chronic pain was measured using a validated item that asked whether participants had experienced physical pain lasting six months or more (yes/no) [34]. Those who responded “yes” were further asked to rate the intensity of their pain over the past four weeks. Responses were combined to create a composite pain score ranging from 1 (no pain) to 5 (very strong pain). NCDs were assessed using a question from the Nord-Trøndelag Health Study (HUNT), asking if participants have any of the following conditions: cardiovascular disease, chronic pulmonary disorder, cancer, or diabetes, with response categorized as “yes/no” [35]. Mental health was measured via symptoms of anxiety/depression and PTSD. Anxiety/depression was assessed using the 10-item Hopkins Symptom Checklist (HSCL-10) [36], which showed good internal consistency (α = 0.87). PTSD symptoms were measured using 16 items from the Harvard Trauma Questionnaire (HTQ-16) [37], demonstrating excellent internal consistency (α = 0.91).

Participants’ age (continuous), gender (female vs. male [reference]), education (years of schooling, continuous), marital status (married vs. others), and parental status (having children vs. none) were measured at the time of exposure (Wave-2).

### Statistical analysis

Continuous variables were summarized using medians and interquartile ranges (IQR), while categorical variables were described using counts and proportions. There was < 5% of data missing for all the variables included. Pearson’s correlation coefficient with p-values was utilized to describe the correlation between sociodemographic factors, health status and integration.

Respondents in Wave-3 were slightly older (mean age: 34.7 vs. 33.9, *p* = 0.461) and included a lower proportion of women (47.7% vs. 51.7%, *p* = 0.410) compared to those in Wave-1 (Table S1). To account for these differences, inverse probability weights based on age and gender distributions from Wave-1 were applied.

We used hierarchical linear regression to examine associations between self-reported health status and integration outcomes (overall and by dimension). In Step 1, demographic variables (age, gender, and education) were entered. Marital status and having children were excluded as their inclusion did not improve model fit. In Step 2, health-related variables, including self-reported chronic pain, NCDs, anxiety/depression and PTSD scores, were added to assess the extent to which health predicted integration difficulties. All models were estimated using ordinary least squares regression with robust standard errors and incorporated the applied weights. Model fit and the incremental explanatory power of each step were assessed via changes in R² (ΔR²), and partial F-tests evaluated the joint significance of added variables.

We did two sensitivity analysis. First, poor health was redefined using dichotomous indicators based on validated cut-offs (HSCL-10 ≥ 1.85 for anxiety/depression [36], HTQ ≥ 2.5 for PTSD [37], and yes/no for chronic pain [34]). Second, cross-sectional associations between health and integration were examined using data only from Wave-3.

All statistical analyses were conducted using Stata, version 19 (StataCorp, College Station, TX).

## Results

A total of 132 participants completed both follow up surveys in Norway. Table 1 summarizes the baseline characteristics of the study sample. The median age was 38 (31–43) years, with 45% female participants. The median duration of formal education was 7 (6–10) years. Most were married and had children. One year after arrival, 10% of participants reported at least one NCDs, one-third experienced some degree of chronic pain, and 14% and 3% reported symptoms of anxiety/depression and PTSD, respectively.

**Table 1:** Study population characteristics at one-year post-resettlement, *N* = 132

	*n*	%
Gender		
Male	72	55
Female	59	45
Age in years, median (IQR)	38	31–43
Education in years, median (IQR)	7	6–10
Marital status		
Married	97	73
Had children		
Yes	110	83
Children number, median (IQR)	4	3–5
Non-communicable diseases ^a^		
Yes	13	10
Chronic pain		
No	88	68
Mild	8	6
Moderate	16	12
Strong	11	8
Very strong	8	6
Anxiety/depression (HSCL-10 cut-off 1.85) ^b^	19	14
Post-traumatic stress disorder (HTQ cut-off 2.5) ^c^	4	3

^a^ Respondent’s with any one of the following complications: cardiovascular disease, chronic pulmonary disorder, cancer, or diabetes

^b^ Hopkins Symptoms Checklist (HSCL-10)

^c^ Harvard Trauma Questionnaire

Four years after resettlement, participants reported the greatest difficulties in social and linguistic integration (Table S2). All dimensions of integration were positively correlated, although generally modestly, with the strongest association observed between linguistic and social integration (*r* = 0.41).

Table 2 presents the correlations between health in the early resettlement phase and later integration outcomes. Chronic pain was negatively correlated with economic integration. Having NCDs was negatively correlated with overall integration, specifically social and linguistic integration. Symptoms of anxiety/depression and PTSD were both negatively correlated with overall integration and with most individual dimensions.

**Table 2:** Pearson’s correlation coefficients for study variables

Factors	Overall integration	Social integration	Psychological integration	Economic integration	Linguistic integration	Navigational integration
Age	-0.44***	-0.28**	-0.11	-0.21*	-0.65***	0.02
Female	-0.08	0.10	-0.10	-0.03*	-0.03	-0.21*
Education	0.17*	0.14	0.03	0.02	0.26**	-0.02
Chronic pain ^a^	-0.12	-0.06	-0.03	-0.28**	-0.11	0.06
NCDs ^b^	-0.19*	-0.23**	-0.12	-0.12	-0.45***	0.08
Anxiety/depression ^c^	-0.35***	-0.21*	-0.18*	-0.18*	-0.31***	-0.12
PTSD ^d^	-0.21*	-0.13	-0.12	-0.20	-0.15	-0.01

*p < 0.05, **p < 0.01, ***p < 0.001

^a^ Pain score on a scale from 1 (no pain) to 5 (very strong pain)

^b^ Non communicable disease, respondents with any one of the following complications: cardiovascular disease, chronic pulmonary disorder, cancer, or diabetes

^c^ Hopkins Symptom Checklist (HSCL-10)

^d^ Post-traumatic stress disorder score, using Harvard Trauma Questionnaire

Table 3 shows result from a two-step hierarchical linear regression examining whether health in early- post-resettlement period predicts later integration outcomes among Syrian refugees in Norway. Changes in individual covariate estimates at each step are shown in Table S3. In Step 1, older age at arrival was associated with overall integration difficulties, particularly within the social, linguistic, and economic dimensions. Female refugees were more likely to encounter challenges in navigational integration, while higher education predicted better linguistic integration. In Step 2, the addition of health-related variables significantly improved the model fit for overall integration measured four years after arrival (R² = 0.286, adjusted R² = 0.086, *p* < 0.001). Symptoms of anxiety/depression reported one year after resettlement were associated with increased overall integration difficulties at follow-up.

**Table 3:** Hierarchical linear regression analysis of predictors of integration

Outcome	Predictors		Standardized coefficient	Standard error	*P*-value	*P*	R2	∆R2
Overall integration	Step 1	Age	-0.412	0.001	< 0.001	< 0.001	0.199	
		Female	-0.041	0.023	0.617			
		Education	0.107	0.005	0.196			
	Step 2	Chronic pain ^a^	0.134	0.010	0.139	< 0.01	0.286	0.086
		NCD ^b^	0.054	0.052	0.605			
		Anxiety/depression ^c^	-0.324	0.038	0.010			
		PTSD ^d^	-0.034	0.031	0.730			
Social integration	Step 1	Age	-0.259	0.002	0.002	0.035	0.087	
		Female	0.133	0.047	0.129			
		Education	0.103	0.008	0.298			
	Step 2	Chronic pain ^a^	0.107	0.022	0.319	0.293	0.133	0.046
		NCD ^b^	-0.077	0.096	0.451			
		Anxiety/depression ^c^	-0.133	0.073	0.289			
		PTSD ^d^	-0.094	0.063	0.366			
Psychological integration	Step 1	Age	-0.094	0.002	0.356	0.454	0.025	
		Female	-0.069	0.032	0.447			
		Education	0.017	0.005	0.864			
	Step 2	Chronic pain ^a^	0.058	0.013	0.524	0.073	0.085	0.060
		NCD ^b^	0.216	0.059	0.023			
		Anxiety/depression ^c^	-0.212	0.058	0.161			
		PTSD ^d^	-0.028	0.050	0.823			
Linguistic integration	Step 1	Age	-0.636	0.002	< 0.001	< 0.001	0.444	
		Female	-0.013	0.036	0.851			
		Education	0.158	0.006	0.031			
	Step 2	Chronic pain ^a^	0.203	0.019	0.032	< 0.01	0.538	0.094
		NCD ^b^	-0.172	0.058	0.009			
		Anxiety/depression ^c^	-0.153	0.059	0.156			
		PTSD ^d^	-0.110	0.043	0.145			
Economic integration	Step 1	Age	-0.211	0.002	0.004	0.046	0.046	
		Female	-0.027	0.052	0.814			
		Education	-0.017	0.008	0.848			
	Step 2	Chronic pain ^a^	-0.241	0.027	0.050	0.081	0.138	0.092
		NCD ^b^	-0.012	0.072	0.869			
		Anxiety/depression ^c^	-0.009	0.087	0.949			
		PTSD ^d^	-0.143	0.075	0.214			
Navigational integration	Step 1	Age	0.052	0.003	0.574	0.064	0.058	
		Female	-0.220	0.064	0.015			
		Education	-0.021	0.009	0.807			
	Step 2	Chronic pain ^a^	-0.148	0.028	0.132	0.027	0.156	0.098
		NCD ^b^	0.133	0.112	0.132			
		Anxiety/depression ^c^	-0.419	0.120	0.007			
		PTSD ^d^	0.317	0.099	0.009			

^a^ Pain score on a scale from 1 (no pain) to 5 (very strong pain)

^b^ Non communicable disease, respondents with any one of the following complications: cardiovascular disease, chronic pulmonary disorder, cancer, or diabetes

^c^ Hopkins Symptom Checklist (HSCL-10)

^d^ Post-traumatic stress disorders, using Harvard Trauma Questionnaire

Health in the early post-resettlement period was also associated with individual dimensions of integration. Symptoms of anxiety/depression assessed one year after resettlement were associated with greater difficulties in navigational integration, while PTSD symptoms, surprisingly, were positively associated with better navigational integration. Chronic pain was associated with subsequent challenges in economic integration but also with better linguistic integration. NCDs were associated with poorer linguistic integration yet better psychological integration.

Findings from sensitivity analyses using validated dichotomous health thresholds were in line with the main findings (results not shown). Consistent associations were also observed in cross-sectional analyses using Wave-3 data (results not shown).

## Discussion

### Main findings

In this prospective cohort study of Syrian refugees in Norway, health status shortly after arrival was associated with integration outcomes four year later with different health problems specifically linked to some domains of integration. Poor health was generally associated with greater challenges across several domains: chronic pain was linked to economic strain, NCDs to language-related barriers, and symptoms of poor mental health to difficulties in overall integration, particularly in navigating the healthcare system. However, poorer health did not uniformly predict disadvantage and was occasionally associated with fewer challenges in specific areas, indicating a more complex, domain-dependent relationship. These findings point to early health as an important factor influencing diverse integration pathways. Given the study’s observational nature and modest sample size, the results should be interpreted with caution, yet they support the need to incorporate health considerations into refugee integration policies.

Four years after resettlement, Syrian refugees in Norway reported varied integration experiences. Many reported a strong sense of belonging and relative ease accessing healthcare yet building social connection and acquisition of Norwegian language remained major challenges. This highlights that progress in one integration domain does not guarantee success in others. Compared to recent cross-sectional studies among Syrian refuges in Norway [5, 38], our sample demonstrated higher levels of psychological and economic integration, likely due to their younger age profile and longer length of stay, unlike other studies with more varied durations of residence. The broader definition of employment used in our study may also have contributed to observed higher economic integration scores. Despite these positive signs, only about one-quarter of participants were employed after four years, indicating persistent barriers to labour market participation. Such barriers may reflect limitations of the Introduction Programme, which has been noted to cultivate primarily horizontal social capital, connections within refugee communities, rather than vertical social capital, which may be more relevant for accessing employment [39]. Additionally, labour market conditions during the study period were influenced by global events such as the COVID-19 pandemic and the war in Ukraine, likely further limiting employment opportunities. Experiences of discrimination may also impede labour market participation and erode trust in public institutions [33].

Health is a vital resource that enables refugees to participate meaningfully in their new society [10]. Our study found that poor health during the early resettlement phase was generally associated with later integration difficulties. Poor physical health, particularly severe chronic pain and NCDs, was associated with later difficulties in securing employment and learning the language, both key components of integration [15]. Although symptoms of poor mental health were less prevalent in our sample than in many studies of newly resettled refugees [17, 40, 41], possibly reflecting a temporary “honeymoon” phase [20], individuals reporting symptoms of anxiety/depression experienced poorer outcomes across all five integration domains including employment, language acquisition, social connections, sense of belonging, and access to health services. At the domain level, anxiety/depression were most strongly associated with difficulties navigating the healthcare system, which may delay timely and appropriate care. Conversely, PTSD symptoms were positively associated with navigational integration; however, this result should be interpreted cautiously due to the small number of participants meeting clinical PTSD threshold (Table S1). It is possible that individuals with more severe symptoms are more likely to be identified and supported by the healthcare system, receiving treatments that aid their integration despite their greater health challenges.

Interestingly, and contrary to some previous research [5], our findings suggest that poor health may, in certain domains, be linked to less integration difficulties, highlighting the complex and non-linear nature of the integration process. Refugees living with NCDs reported a stronger sense of belonging and felt less like outsiders four years after resettlement, indicating more positive psychological integration. This may be explained by the comprehensive support offered by the Norwegian healthcare system for chronic conditions, which includes regular medical follow-ups and subsidized treatments— services that likely contrast sharply with refugees’ experiences in conflict-affected regions like Syria [42]. In contrast, symptoms of poor mental health were not associated with increased sense of connectedness. Although mental health difficulties can influence perceptions of social support and circumstances, sensitivity analyses excluding participants with poor mental health at the time of assessment did not change the results. This may indicate ongoing gaps in mental health support [26], but further research is necessary. Additionally, refugees reporting chronic pain were predominantly younger, which might partly explain their comparatively better language integration, as younger individuals generally learn new languages more rapidly. Due to the small sizes of these subgroups, these findings should be interpreted with caution, underscoring the complex and sometimes unexpected ways in which health influences integration.

Support programs such as the Introduction Programme play an important role in refugee integration by providing essential knowledge for navigating institutions and daily life in the host country, as well as opportunities to build social networks [23], regardless of health status. Addressing health needs alongside these supports has been shown to enhance the effectiveness of such initiatives and related interventions [43]. Accessible and culturally sensitive healthcare during early resettlement is therefore important not only for managing immediate health concerns but also for supporting refugees’ engagement with social and welfare systems. General practitioners are the primary point of contact in healthcare, playing a central role in delivering frontline care and connecting refugees to broader welfare and support services [7]. However, Syrian refugees in Norway often face barriers to accessing such support, especially language difficulties [16]. Although interpreter services are provided free of charge, their inconsistent availability and lack of proactive coordination [44] can impede effective communication and potentially compromise care quality [45, 46]. Furthermore, many healthcare providers report feeling inadequately prepared to address refugees’ complex health needs [47], which may contribute to delays in care, diminished trust in the healthcare system, and poorer health outcomes [7].

### Implications

While the number of refugees worldwide soars, it is increasingly important to understand the factors that support successful integration [8]. Although resettlement offers safety, the early period poses significant challenges as refugees navigate multiple stressors [5]. In line with Ager and Strang’s framework [10], our findings support health as a *means* that enables integration across several domains, highlighting the need to consider health alongside other core elements of resettlement support. Research suggests that Syrian refugees often experience worsening health outcomes with longer stays in Norway [25]. Therefore, support programs should be regularly evaluated and adapted to meet refugees’ evolving needs and lived experiences. Ensuring such flexibility requires strong cross-sectoral collaboration among health, social services, education, and employment sectors [23].

Within this broader context, ensuring continuity of care and fostering relationships between healthcare providers and refugees is a key priority [7]. Strengthening cultural competence among healthcare professionals may further improve the quality and responsiveness of care [44], particularly for refugees with intertwined physical- and mental health needs [48]. Recognizing and strengthening the bridging role of general practitioners within the healthcare system as part of cross-sectoral integration strategies may enable refugees to navigate services more effectively and engage more fully in Norwegian society [7]. Embedding these priorities within integration efforts can ultimately support better health outcomes and facilitate successful resettlement.

### Strength and limitations

A key strength of this study is its longitudinal, prospective design, which enabled examination of the association between early post-resettlement health and later integration outcomes. The use of a validated and standardized measurement tool allowed for assessment of both overall and domain-specific integration scores, providing a nuanced understanding of refugees’ engagement and inclusion within the host society. Additionally, the IPL’s brevity makes it suitable for use in surveys with the so-called “hard-to-reach populations”, such as refugees [16].

This study has several limitations. Integration was assessed across five dimensions using the IPL Index, excluding the political domain. Due to low inter-item correlations, single items were used for the economic and navigational dimensions, and the psychological and social subscales showed reliability coefficients below conventional thresholds (α < 0.70) [49]. Nonetheless, these domains correlated meaningfully with other dimensions of integration and health parameters, supporting their validity. Health measures were based on self-reported symptoms rather than clinical diagnoses. There was substantial loss to follow-up over time: the initial survey (Wave-1) included 97% of Syrian refugees resettled at that time [20], but participation fell to 33% by Wave-3, which could introduce selection bias. However, sensitivity analyses comparing key characteristics between participants included in the study and those lost to follow-up found no significant differences (Table S4), suggesting that attrition likely had minimal impact on our results. The study period coincided with the COVID-19 pandemic, which may have unevenly influenced integration experiences. Our study focuses exclusively on Syrian refugees, which may limit the generalizability of the findings to other refugee populations. While some experiences are likely shared across groups, the complex interplay of factors such as gender, education, religion, and other unmeasured variables related to intersectionality need to be considered. Finally, the four-year follow-up may not fully capture the long-term relationship between health and integration.

## Conclusion

Among Syrian refugees four years after resettlement in Norway, poor health shortly after arrival was mostly associated with greater challenges across multiple domains of integration. Specifically, chronic pain was linked to economic strain, NCDs to language-related barriers, and symptoms of poor mental health to difficulties in overall integration, particularly in navigating the healthcare services. These findings underscore the importance of timely and culturally sensitive healthcare as part of broader efforts to support refugee integration.

## Supplementary Information

Below is the link to the electronic supplementary material.

Supplementary Material 1

## Data Availability

The datasets used in this study are not publicly available and can only be accessed upon reasonable request, subject to approval by the Regional Committees for Medical and Health Research Ethics (REK) and the University of Bergen. Data access requests should be directed to esperanza.diaz@uib.no.

## Abbreviations

CI: Confidence Interval
HSCL: Hopkins Symptom Checklist
HTQ: Harvard Trauma Questionnaire
IPL: Immigration Policy Lab
NCDs: Non-communicable diseases
PTSD: Post-traumatic distress disorders
RR: Relative Risk
