# Supplementary material for: Does health early at arrival predict later integration among refugees? A cohort study of Syrians in Norway
Source: Int J Equity Health. 2026 Apr 7;25:91. doi: 10.1186/s12939-026-02837-8 (PMC13067588; doi:10.1186/s12939-026-02837-8)
Supplement: Supplementary file 1 — Supplementary Material 1 [file 12939_2026_2837_MOESM1_ESM.docx]

**Supplementary Material**

**Table S1**. Characteristics of Participants in the CHART/Integration for Health Project across Waves 1–3 (2017–2023)

|  | **Wave-1, 2017-2018 (N = 506)** | **Wave-2, 2018-2019**  **(N = 353)** | **Wave-3, 2022-2023**  **(N = 153)** |
| --- | --- | --- | --- |
|  | **n (%)** | **n (%)** | **n (%)** |
| **Sociodemographic factors** |  |  |  |
| Gender |  |  |  |
| Male | 250 (49) | 171 (48) | 80 (52) |
| Female | 256 (51) | 182 (52) | 73 (48) |
| Age (years), mean (SD) | 34 (11) | 34 (11) | 35 (11) |
| Education (years), mean (SD) | 8 (3) | 8 (3) | 8 (4) |
| **Health-related factors** |  |  |  |
| Chronic pain |  |  |  |
| No | 360 (71) | 253 (73) | 75 (49) |
| Mild | 28 (6) | 21 (6) | 12 (8) |
| Moderate | 59 (12) | 34 (10) | 22 (14) |
| Strong | 34 (7) | 29 (8) | 33 (21) |
| Very strong | 17 (3) | 11 (3) | 12 (8) |
| Non-communicable diseases ^a^ |  |  |  |
| Yes | 59 (12) | 29 (8) | 38 (24) |
| Anxiety/depression (HSCL-10 cut-off 1.85) ^b^ | 178 (35) | 39 (11) | 45 (29) |
| Post-traumatic stress disorder (HTQ cut-off 2.5) ^c^ | 26 (5) | 7 (2) | 7 (5) |

^a^ Respondent’s with any one of the following conditions: cardiovascular disease, chronic pulmonary disorder, cancer, or diabetes

^b^ Hopkins Symptom Checklist-10 above threshold

^c^ Harvard Trauma Questionnaire above threshold

**Table S2**. Descriptive statistics and correlation matrix for overall and individual dimensions of integration, measured using the Immigration Policy Lab Integration Index.

|  | Mean | Median | IQR | Cronbach alpha | Overall integration | Social integration | Psychological integration | Economic integration | Linguistic integration | Navigational integration |
| --- | --- | --- | --- | --- | --- | --- | --- | --- | --- | --- |
| Overall integration | 0.60 | 0.61 | 0.51-0.70 | NA^a^ | 1.00 |  |  |  |  |  |
| Social integration | 0.47 | 0.50 | 0.25-0.63 | 0.60 | 0.70*** | 1.00 |  |  |  |  |
| Psychological integration | 0.68 | 0.69 | 0.56-0.81 | 0.61 | 0.69*** | 0.27** | 1.00 |  |  |  |
| Economic integration | 0.66 | 0.75 | 0.38-1.00 | NA | 0.39*** | 0.22* | 0.02 | 1.00 |  |  |
| Linguistic integration | 0.53 | 0.50 | 0.38-0.75 | 0.77 | 0.58*** | 0.41*** | 0.09 | 0.13 | 1.00 |  |
| Navigational integration | 0.64 | 0.75 | 0.25-1.00 | NA | 0.26** | -0.11 | 0.17* | 0.02 | -0.11 | 1.00 |

*p<0.05, **p<0.01, ***p<0.001

^a^ Not applicable

**Table S3. Hierarchical linear regression analysis of predictors of integration**

| **Outcome** | **Predictors** | | **Standardized coefficient** | **Standard error** | ***P*-value** | ***P*** | **R2** | **∆R2** |
| --- | --- | --- | --- | --- | --- | --- | --- | --- |
| Overall integration | Step 1 | Age | -0.412 | 0.001 | < 0.001 | <0.001 | 0.199 |  |
|  |  | Female | -0.041 | 0.023 | 0.617 |  |  |  |
|  |  | Education | 0.107 | 0.005 | 0.196 |  |  |  |
|  | Step 2 | Age | -0.383 | 0.001 | < 0.001 | <0.01 | 0.286 | 0.086 |
|  |  | Female | -0.045 | 0.023 | 0.585 |  |  |  |
|  |  | Education | 0.107 | 0.003 | 0.183 |  |  |  |
|  |  | Chronic pain ^a^ | 0.134 | 0.010 | 0.139 |  |  |  |
|  |  | NCD ^b^ | 0.054 | 0.052 | 0.605 |  |  |  |
|  |  | Anxiety/depression ^c^ | -0.324 | 0.038 | 0.010 |  |  |  |
|  |  | PTSD ^d^ | -0.034 | 0.031 | 0.730 |  |  |  |
| Social integration | Step 1 | Age | -0.259 | 0.002 | 0.002 | 0.035 | 0.087 |  |
|  |  | Female | 0.133 | 0.047 | 0.129 |  |  |  |
|  |  | Education | 0.103 | 0.008 | 0.298 |  |  |  |
|  | Step 2 | Age | -0.209 | 0.003 | 0.044 | 0.293 | 0.133 | 0.046 |
|  |  | Female | 0.113 | 0.049 | 0.210 |  |  |  |
|  |  | Education | 0.104 | 0.009 | 0.323 |  |  |  |
|  |  | Chronic pain ^a^ | 0.107 | 0.022 | 0.319 |  |  |  |
|  |  | NCD ^b^ | -0.077 | 0.096 | 0.451 |  |  |  |
|  |  | Anxiety/depression ^c^ | -0.133 | 0.073 | 0.289 |  |  |  |
|  |  | PTSD ^d^ | -0.094 | 0.063 | 0.366 |  |  |  |
| Psychological integration | Step 1 | Age | -0.094 | 0.002 | 0.356 | 0.454 | 0.025 |  |
|  |  | Female | -0.069 | 0.032 | 0.447 |  |  |  |
|  |  | Education | 0.017 | 0.005 | 0.864 |  |  |  |
|  | Step 2 | Age | -0.182 | 0.002 | 0.104 | 0.073 | 0.085 | 0.060 |
|  |  | Female | -0.034 | 0.034 | 0.715 |  |  |  |
|  |  | Education | 0.034 | 0.005 | 0.739 |  |  |  |
|  |  | Chronic pain ^a^ | 0.058 | 0.013 | 0.524 |  |  |  |
|  |  | NCD ^b^ | 0.216 | 0.059 | 0.023 |  |  |  |
|  |  | Anxiety/depression ^c^ | -0.212 | 0.058 | 0.161 |  |  |  |
|  |  | PTSD ^d^ | -0.028 | 0.050 | 0.823 |  |  |  |
| Linguistic integration | Step 1 | Age | -0.636 | 0.002 | < 0.001 | <0.001 | 0.444 |  |
|  |  | Female | -0.013 | 0.036 | 0.851 |  |  |  |
|  |  | Education | 0.158 | 0.006 | 0.031 |  |  |  |
|  | Step 2 | Age | -0.576 | 0.002 | < 0.001 | <0.01 | 0.538 | 0.094 |
|  |  | Female | -0.044 | 0.034 | 0.505 |  |  |  |
|  |  | Education | 0.143 | 0.005 | 0.037 |  |  |  |
|  |  | Chronic pain ^a^ | 0.203 | 0.019 | 0.032 |  |  |  |
|  |  | NCD ^b^ | -0.172 | 0.058 | 0.009 |  |  |  |
|  |  | Anxiety/depression ^c^ | -0.153 | 0.059 | 0.156 |  |  |  |
|  |  | PTSD ^d^ | -0.110 | 0.043 | 0.145 |  |  |  |
| Economic integration | Step 1 | Age | -0.229 | 0.002 | 0.004 | 0.049 | 0.046 |  |
|  |  | Female | -0.021 | 0.052 | 0.814 |  |  |  |
|  |  | Education | -0.017 | 0.008 | 0.848 |  |  |  |
|  | Step 2 | Age | -0.151 | 0.002 | 0.104 |  |  |  |
|  |  | Female | -0.009 | 0.516 | 0.919 |  |  |  |
|  |  | Education | -0.020 | 0.007 | 0.812 |  |  |  |
|  |  | Chronic pain ^a^ | -0.241 | 0.027 | 0.050 | 0.081 | 0.138 | 0.092 |
|  |  | NCD ^b^ | -0.012 | 0.072 | 0.869 |  |  |  |
|  |  | Anxiety/depression ^c^ | -0.009 | 0.087 | 0.949 |  |  |  |
|  |  | PTSD ^d^ | -0.143 | 0.075 | 0.214 |  |  |  |
| Navigational integration | Step 1 | Age | 0.052 | 0.003 | 0.574 | 0.064 | 0.058 |  |
|  |  | Female | -0.220 | 0.064 | 0.015 |  |  |  |
|  |  | Education | -0.021 | 0.009 | 0.807 |  |  |  |
|  | Step 2 | Age | 0.085 | 0.032 | 0.371 | 0.027 | 0.156 | 0.098 |
|  |  | Female | -0.225 | 0.065 | 0.013 |  |  |  |
|  |  | Education | -0.025 | 0.009 | 0.747 |  |  |  |
|  |  | Chronic pain ^a^ | -0.148 | 0.028 | 0.132 |  |  |  |
|  |  | NCD ^b^ | 0.133 | 0.112 | 0.132 |  |  |  |
|  |  | Anxiety/depression ^c^ | -0.419 | 0.120 | 0.007 |  |  |  |
|  |  | PTSD ^d^ | 0.317 | 0.099 | 0.009 |  |  |  |

**Table S4.** Sociodemographic factors among participants included at three data collection points compared to those lost to follow-up during first (Wave-2) or second (Wave-3) follow-up in Norway

|  | **Participated in Wave-2 and Wave-3** | **Lost to follow up at**  **Wave-3** | **p-value^a^** |
| --- | --- | --- | --- |
|  | **n (%)** | **n (%)** |  |
| N | 132 | 353 |  |
| Age at Wave-1, mean (SD) | 35 (11) | 34 (13) | 0.63 |
| Gender |  |  |  |
| Men | 73 (55) | 182 (52) | 0.66 |
| Women | 59 (45) | 170 (48) |  |
| Education at Wave-1, mean (SD) | 8 (3) | 8 (4) | 0.53 |

^a^ t-test for continuous variables, chi-square test for categorial variables
